# Extracellular Vesicles in Angelman Syndrome: Expanding UBE3A Role beyond a Cell Autonomous Mechanism

**Published:** 2026

**Authors:** Eduardo Penna, Michel Baudry, Xiaoning Bi

**Affiliations:** 1 Department of Biology, University of Naples Federico II, Naples, Italy; 2 College of Dental Medicine, Western University of Health Sciences, Pomona, CA, USA; 3 College of Osteopathic Medicine of the Pacific, Western University of Health Sciences, Pomona, CA, USA

**Keywords:** Angelman syndrome, Autonomous mechanism, Neurodevelopmental disorder

## Abstract

Angelman syndrome (AS) is a severe neurodevelopmental disorder caused by the loss of maternal UBE3A, an E3 ubiquitin ligase essential for neuronal development, synaptic plasticity, and cognitive function. AS has traditionally been viewed as a cell-autonomous disorder in which intracellular UBE3A deficiency drives neuronal dysfunction. However, recent evidence suggests that impaired extracellular vesicle (EV)-mediated intercellular communication also contributes to disease pathophysiology. EVs are key intercellular mediators of neuronal and glial signaling, as they transfer proteins, lipids, and nucleic acids, which regulate synaptic homeostasis, circuit maturation, and brain plasticity. Here, we discuss emerging evidence linking UBE3A deficiency to alterations in EV biogenesis, cargo composition, secretion, and uptake. We propose that disruption of ubiquitin-dependent endosomal trafficking, together with endolysosomal dysfunction involving LAMTOR1 and the lysosomal Ca^2+^ channel TRPML1, contribute to defective EV properties in AS. Moreover, the recent identification of UBE3A within neuronal EVs raises the possibility that loss of EV-mediated UBE3A transfer represents an additional non-cell-autonomous mechanism underlying synaptic dysfunction in AS. These findings support a revised model in which impaired EV signaling amplifies the consequences of intracellular UBE3A deficiency. Finally, we discuss the therapeutic potential of EVs. The ability of wild-type neuron-derived EVs to rescue synaptic and cognitive deficits in an AS mouse model highlights the use of EVs as a promising therapeutic avenue for Angelman syndrome and other neurodevelopmental disorders.

## Extracellular Vesicle (EV) Heterogeneity: Type, Biogenesis and Cargo

EVs represent a heterogeneous population of particles released by almost all cell types; they are involved in intercellular communication under both physiological and pathological conditions [1–4]. Due to their ability to travel in biological fluids and to cross physiological barriers, EVs mediate both local and long-distance cell-to-cell communication [4]. Based on their biogenesis, EVs are commonly categorized into exosomes and microvesicles, although the precise determination of vesicle origin remains challenging [1,2,5].

Microvesicles are relatively large EVs (100–1000 nm) generated by direct outward budding of the plasma membrane and subsequent detachment. This process is calcium-dependent, requires cytoskeletal remodeling, and involves phospholipid redistribution [1]. In contrast, exosomes are small EVs (30–150 nm) originating from the endosomal system through inward budding of multivesicular body (MVB) membranes, which generate intraluminal vesicles (ILVs). Following MVB fusion with the plasma membrane, ILVs are released extracellularly as exosomes [3,4,6]. This mechanism is regulated by the machinery of the endosomal sorting complex required for transport (ESCRT), which orchestrates membrane deformation and scission events through the coordinated action of ESCRT-0, -I, -II and -III complexes [7,8]. However, exosomes also originate trough ESCRT-independent mechanisms, involving lipid-driven membrane curvature mediated by ceramide, cholesterol-enriched microdomains, and tetraspanin-enriched domains [4,9].

Lysosomes also participate in EV release through lysosomal exocytosis, leading to the secretion of lysosomal content [10,11]. This process is regulated by the lysosomal transient receptor potential mucolipin 1 (TRPML1), a channel mediating lysosomal Ca^2+^ release [11–13]. Thus, lysosomes represent a key regulator of EV biogenesis and secretion. Indeed, lysosomal dysfunction has been shown to increase EV release in several cellular systems, suggesting that EV secretion may serve as an alternative clearance route when degradative capacity is compromised [14].

EVs regulate target cells by transporting and transferring a variety of materials, such as DNA, RNA, lipids, and proteins [4]. Cargo loading is not a random event, but a selective process governed by specific proteins, lipid modifications, and intracellular signals that determine which molecules are incorporated into vesicles [4,15]. For instance, the ESCRT machinery recognizes ubiquitinated proteins and sorts them into forming vesicles, whereas ceramides and tetraspanins such as CD63 and CD81 create membrane microdomains capable of attracting specific proteins and excluding others. Furthermore, RNA-binding proteins recognize and guide mRNA or miRNA molecules into forming vesicles [16].

EV cargo composition reflects vesicle biogenesis. Microvesicles largely retain plasma membrane-associated proteins, lipids, and cytosolic components, whereas exosomes are enriched in endosomal proteins, including the tetraspanins CD9, CD63, and CD81, together with the ESCRT-associated cytosolic proteins ALIX and TSG101[1,3]. Accumulating evidence indicates that the classical tetraspanins CD9, CD63, and CD81 are not uniformly distributed across all EVs. Instead, distinct EV subpopulations display unique tetraspanin profiles, reflecting differences in their intracellular origin and biological functions [17,18]. Accordingly, combinations of tetraspanin markers may be useful for identifying and characterizing specific EV subpopulations rather than defining a single homogeneous exosome population.

Collectively, EV cargo sorting is a highly regulated and selective process, which generates vesicles reflecting both the molecular identity and the physiological state of donor cells. Because cargo loading is dynamically modulated by stress, inflammation, metabolic alterations, senescence, and lysosomal dysfunction, EV composition can change substantially, resulting in functionally distinct vesicle populations with specific molecular signatures and biological activities. Taken together, EV biogenesis and cargo selection emerge from a coordinated interplay among endosomal trafficking, membrane lipid organization, and lysosomal function, ultimately shaping EV composition, heterogeneity, and biological activity.

## EV Functions in the CNS: Physiological and Pathological Evidence

The central nervous system (CNS) comprises a highly specialized communication network in which neurons and glial cells continuously exchange molecular information to maintain neuronal homeostasis and synaptic function. Emerging evidence indicates that EVs contribute to this communication, acting alongside neurotransmitters, soluble factors, and direct cell-cell interactions to coordinate neuronal and glial functions [19,20]. Accordingly, EVs participate in multiple aspects of brain physiology and also contribute to various diseases when their production, cargo composition, or cellular uptake is dysregulated.

### Physiological functions of EVs in the CNS

Within the CNS, EVs contribute to neuronal communication, synaptic plasticity, and metabolic support [21,22]. Importantly, EV-mediated communication is not restricted to similar cell types; rather, EVs function as versatile messengers, which facilitate cross-talk among diverse cell populations. Neurons, astrocytes, oligodendrocytes, and microglia release distinct EV populations carrying cell-specific molecular cargoes, which reflect their specialized roles within the brain [23–25].

Specifically, neuronal EVs help maintain neuronal structure by promoting spine formation and preserving neuronal complexity [26]. Glial-derived EVs are equally critical: astrocyte-derived EVs provide metabolic support and regulate local protein synthesis, oligodendrocyte-derived EVs promote axonal resilience under stress, and microglial EVs participate in synaptic remodeling, circuit refinement, and immune surveillance [24,25,27]. Through these exchanges, EVs coordinate neuronal and glial functions, maintaining CNS homeostasis, synaptic integrity, and circuit stability.

A key feature of neuron-derived EV biology is its sensitivity to neuronal activity. EV secretion is dynamically regulated by neuronal stimulation, and depolarization-induced calcium influx increases EV release [28,29]. These findings suggest that EV secretion is an integral component of activity-dependent neuronal signaling, contributing to synaptic adaptation and homeostatic plasticity. Neuronal activity also influences EV cargo composition: brain-derived neurotrophic factor (BDNF) stimulation alters EV cargo composition and promotes the transfer of synaptogenic microRNAs that enhance excitatory synapse formation, increase synaptic vesicle clustering, and facilitate circuit maturation [21]. Together these studies establish EVs as key mediators of neuronal activity-dependent synaptic plasticity and neural circuit remodeling.

The importance of EVs is particularly evident during brain development, when precise and coordinated communication among diverse cell populations is required for neuronal differentiation, synaptogenesis, and circuit maturation. EV cargoes change throughout development, indicating that EV-mediated signaling dynamically adapts to the evolving needs of the developing nervous system. By adapting their signaling properties over time, EVs help establish, refine, and stabilize functional neural circuits [22,30].

Collectively, these findings indicate that EVs are integral components of CNS physiology, serving as key regulators of synaptic and cellular homeostasis.

### EV dysfunction in neurological disorders

Given their central role in intercellular communication, alterations in EV biology have been increasingly implicated in neurological diseases. Dysregulation of EV production, cargo sorting, secretion, or uptake can profoundly affect neuronal and glial functions, ultimately influencing neural circuit integrity.

In neurodegenerative disorders, EVs contribute to pathogenesis by facilitating the transfer of aggregation-prone proteins, including amyloid-beta, tau, and alpha-synuclein, across interconnected brain regions [15,31–33], thereby accelerating disease progression. EVs also participate in neuroinflammatory responses. Under pathological conditions, microglial and astrocytic activation alters EV cargo composition, increasing the transfer of inflammatory mediators and immune signaling molecules [27,34,35]. Consequently, EVs can amplify neuroinflammation, disrupt synaptic function, and exacerbate neuronal dysfunction.

Importantly, EV dysfunction is not limited to neurodegenerative disorders but also contributes to neurodevelopmental diseases. Because EV-mediated signaling supports neuronal maturation and synaptic plasticity, its perturbations during critical developmental windows may have long-lasting consequences for circuit formation and cognitive function [36,37]. Taken together, these findings support a model in which EVs mediate both physiological and pathological communication within the CNS. Understanding how EV signaling is disrupted across neurological disorders may therefore reveal disease mechanisms as well as biomarker and therapeutic opportunities.

## Angelman Syndrome

Angelman syndrome (AS) is a severe neurodevelopmental disorder characterized by intellectual disability, motor dysfunction, epilepsy, and profound deficits in learning and memory [38,39]. AS is caused by loss of function of the maternally inherited UBE3A gene located on chromosome 15 (15q11.2-q13) [39]. In neurons, UBE3A is expressed only from the maternal allele, because the paternal copy of the gene is silenced [40–42]. UBE3A is an E3 ubiquitin ligase that regulates substrate ubiquitination and proteasomal degradation [43]. Importantly, Ube3a plays a key role in dendritic spine maturation, synaptic transmission, activity-dependent plasticity, and experience-dependent circuit refinement [44,45]; disruption of these processes contributes to many of the neurological manifestations of AS.

**Figure 1.**
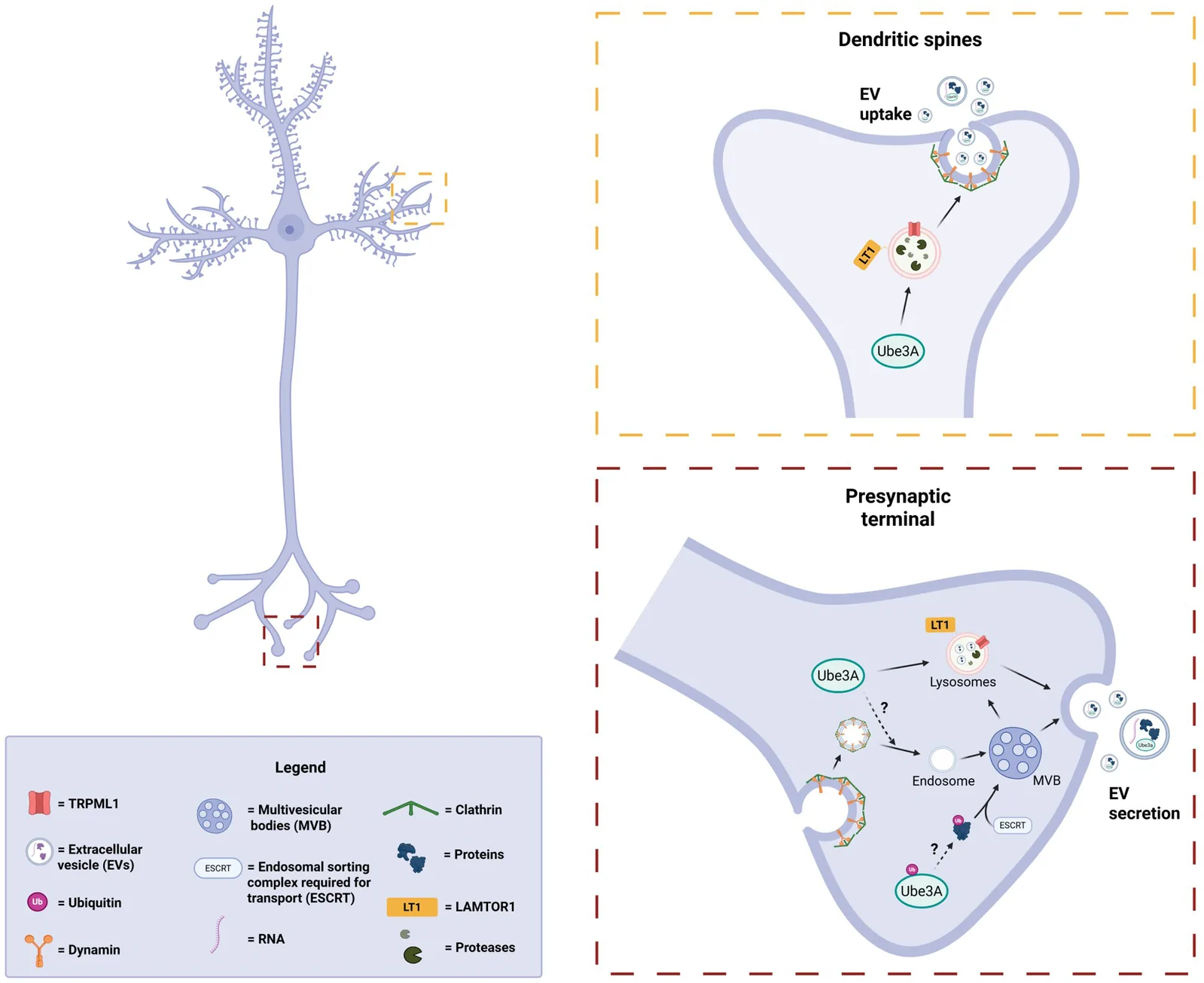
Proposed roles of UBE3A in EV biogenesis, secretion, uptake, and cargo loading. UBE3A mediates ubiquitination and degradation of LAMTOR1, a lysosome-associated protein which tonically inhibit the lysosomal Ca^2+^ channel TRPML1, thereby promoting lysosomal trafficking and EV secretion [51,52]. In Angelman syndrome (AS), loss of UBE3A reduces LAMTOR1 removal, resulting in increased TRPML1 inhibition and defective EV release [11]. UBE3A also appears to regulate EV uptake at dendritic spines through lysosome-dependent mechanisms [11]. In addition, UBE3A contributes to clathrin recycling from endosomal vesicles, a process required for endosomal maturation and multivesicular body (MVB) formation [48]; disruption of this pathway may further reduce EV biogenesis in AS. As an E3 ubiquitin ligase, UBE3A may also participate in ubiquitin-dependent cargo sorting into MVB, thereby influencing EV composition. Finally, the presence of UBE3A within EVs suggests a potential role in EV-mediated intercellular communication [11]. Dotted line: hypothetical involvement of UBE3A. Solid line: direct evidence of UBE3A involvement.

Accordingly, AS has traditionally been viewed as a cell-autonomous disorder in which neuronal dysfunction arises from intracellular molecular abnormalities caused by UBE3A deficiency. However, recent work by Penna and colleagues [11] provides the first evidence that EV dysfunction contributes to synaptic and cognitive deficits in an AS mouse model, expanding the pathogenic framework beyond the established intracellular consequences of UBE3A deficiency. These findings suggest that UBE3A deficiency disrupts not only intracellular signaling but also EV-mediated intercellular communication, which is essential for coordinating neuronal development and circuit function. Given the close relationships among ubiquitin signaling, endosomal trafficking, and EV biogenesis, several molecular pathways could potentially link UBE3A deficiency to altered EV function (Figure 1). The following sections summarize the available evidence and discuss potential mechanisms where direct experimental validation is still lacking.

### Altered ubiquitin signaling in AS may impair EV biogenesis

A major mechanistic convergent point between UBE3A function and EV biology is the role of ubiquitination within the endosomal system, particularly during MVB biogenesis. Ubiquitinated cargo proteins are recognized by ESCRT-0 components and sequestered into ILVs [4,7,8], which are either degraded by lysosomes or released extracellularly as exosomes [4]. Thus, ubiquitin signaling serves as a critical link between intracellular protein clearance and intercellular communication. Based on this framework, loss of UBE3A function could impair EV biogenesis and secretion by altering ubiquitin-dependent endosomal sorting (Figure 1). Along this line, PEG10, a multifunctional RNA-binding protein, has been identified as an UBE3A substrate and is secreted in neuronal EVs [46]. UBE3A deficiency leads to PEG10 accumulation, which could disrupt neuronal migration and contribute to abnormal brain development.

Although direct experimental evidence linking UBE3A to EV biogenesis is lacking, the established role of ubiquitin in endosomal trafficking and MVB sorting [4,7,8] supports the hypothesis that dysregulated ubiquitin signaling may be one of the earliest molecular mechanisms impairing EV-mediated communication in AS.

### UBE3A deficiency impairs EVs secretion

Under physiological conditions, EV secretion is regulated by neuronal activity and intracellular calcium signaling [28,29]. In AS, severe alterations in baseline neuronal activity and calcium homeostasis [47] provide a plausible basis for impaired EV release. Moreover, UBE3A deficiency leads to a marked presynaptic accumulation of clathrin-coated vesicles (CCVs), indicating a severe impairment of clathrin uncoating and subsequent synaptic membrane recycling [48]. Because endocytic trafficking provides membrane components required for the maintenance of early endosomes, late endosomes, and MVBs, it is possible to hypothesize that sequestration of membranes within persistent CCVs caused by UBE3A deficiency reduce the vesicle pool available for endosomal maturation and MVB biogenesis (Figure 1). Although this link remains to be fully established experimentally, defects in clathrin-mediated recycling provide a plausible explanation for altered EV production and release, particularly for exosomes, whose formation depends on endosomal pathways.

### Endolysosomal dysfunction in AS amplifies EV abnormalities

Neurons rely on endolysosomal pathways for synaptic protein turnover, receptor recycling, and membrane homeostasis. In this context, lysosomes act not only as degradative organelles but also as regulators of EV secretion, since MVBs represent a shared intermediate between lysosomal degradation and exosome release. Dysregulation of these pathways has been widely implicated in neurodevelopmental and neurodegenerative disorders [49,50]. Support for the involvement of endolysosomal dysfunction in AS comes from studies of Christianson syndrome, an X-linked neurodevelopmental disorder with overlapping clinical features, caused by mutations in the endosomal ion transporter NHE6 [51]. NHE6 deficiency disrupts endosomal maturation and lysosomal function, leading to progressive neuronal dysfunction. Although EV biology has not been directly investigated, these findings suggest that impaired endolysosomal trafficking may similarly disrupt MVB dynamics and contribute to EV abnormalities in AS.

Consistent with this concept, previous studies have identified the lysosome-associated protein LAMTOR1 as a substrate of UBE3A-mediated ubiquitination and demonstrated that disruption of this pathway contributes to AS pathogenesis [52]. LAMTOR1 tonically inhibits the lysosomal Ca^2+^ channel TRPML1, whose activity is essential for dendritic lysosomal dynamics, synaptic plasticity, learning, and memory [53]. In AS mice, loss of UBE3A causes LAMTOR1 accumulation within synaptic compartments [52], leading to excessive TRPML1 inhibition and subsequent lysosomal dysfunction, a feature associated with several neurodegenerative and neurodevelopmental disorders [54]. Building on these findings, Penna and colleagues demonstrated that TRPML1-mediated EV secretion is impaired in AS neurons and proposed that excessive LAMTOR1-mediated TRPML1 inhibition suppresses lysosomal trafficking and EV secretion (Figure 1).

Intriguingly, lysosomes may also participate in EV uptake. Penna et al. found that TRPML1 inhibition significantly reduced neuronal EV uptake, suggesting that TRPML1 regulates both EV secretion and recipient-cell internalization (Figure 1). Although the mechanisms remain poorly understood, one possibility is that accumulation of undegraded material caused by lysosomal inhibition impairs clathrin-mediated endocytosis, thereby reducing EV internalization [55]. It is noteworthy that when lysosomal function is impaired, cells often exhibit increased EV release as a compensatory mechanism to dispose of accumulated cargoes [14]. In contrast, AS is characterized by reduced EV release [11]. Nevertheless, increased EV size was reported, suggesting a possible compensatory mechanism of accumulation of material within enlarged EVs rather than distribution across a larger EV population. Importantly, AS represents a distinct pathological condition, as UBE3A deficiency does not cause a classical lysosomal storage phenotype but may disrupt endolysosomal trafficking and EV-mediated intercellular communication.

Collectively, these findings support the hypothesis that endolysosomal dysfunction represents a convergent mechanism linking impaired intracellular trafficking with defective EV-mediated intercellular communication in Angelman syndrome. This shift in intercellular trafficking could have a profound impact on neuronal communication, as altered EV secretion can modify extracellular signaling and the propagation of molecular signals, thereby amplifying synaptic dysfunction across neural circuits.

### UBE3A secretion in neuronal EVs

Another major advance in AS pathogenesis is the discovery that UBE3A itself participates in intercellular communication through neuronal EVs. Penna and colleagues demonstrated for the first time that UBE3A is present in EVs released from wild-type mouse synaptosomes and that its secretion is activity-dependent; noticeably, UBE3A is absent from synaptosomal EVs prepared from AS mice (Figure 1) [11].

Although pathological EV alterations are often attributed to broad changes in cargo composition, increasing evidence indicates that the selective loss of specific cargo proteins can also have important functional consequences. For instance, cells selectively exclude oxidized mitochondrial proteins from EVs to prevent the dissemination of pro-inflammatory mitochondrial damage-associated molecular patterns (DAMPs), and disruption of this process promotes inflammation [56]. Similarly, the absence of UBE3A in AS-derived EVs raises the possibility that loss of UBE3A transfer between neighboring cells contributes to impaired intercellular communication in AS, in addition to its intracellular deficiency. For instance, recent evidence suggests that neuronal UBE3A deficiency generates soluble signals, such as TNF and complement components, that trigger microglial activation, inflammatory response and aberrant neuronal synaptic engulfment [57]. Similarly, EVs released by UBE3A-deficient neurons may contain an altered molecular cargo that could influence microglial and astrocytic responses. Accordingly, UBE3A transfer through neuronal EVs may represent a potential mechanism of intercellular protein transfer to glial cells, where UBE3A could contribute to the regulation of protein stability and cellular homeostasis. Loss of EV-mediated signaling pathway may therefore contribute to impaired neuron–glia communication and altered glial functions, including metabolic support and myelination of neuronal circuits [58]. This possibility suggests the hypothesis that an alteration in EV communication between neurons and glial cells may contribute to a feedback loop that further exacerbates synaptic dysfunction in AS.

Beyond mature neural circuits, altered EV signaling may also influence early brain development. Since UBE3A deficiency affects neural progenitor populations [59] and EVs regulate progenitor proliferation, differentiation, and lineage specification [30], impaired EV-mediated communication within the neurogenic niche could contribute to the early developmental abnormalities characteristic of AS. Although these mechanisms remain speculative, they underscore the importance of considering EV heterogeneity—including neuron-, astrocyte-, microglia-, and progenitor-derived EVs—when investigating AS pathogenesis and developing EV-based therapeutic strategies.

Collectively, these observations suggest a model in which UBE3A regulates synaptic and circuit properties not only through its established role in intracellular proteostasis but also by controlling the endosome-lysosome-EV axis. Loss of UBE3A would therefore impair endosomal trafficking, lysosomal function, EV secretion, and EV cargo composition while simultaneously eliminating EV-mediated transfer of UBE3A itself. Together, these mechanisms provide a unified framework in which both cell-autonomous and non-cell-autonomous dysfunction contribute to synaptic and circuit abnormalities in AS.

## EVs as Biomarkers for AS

EVs have emerged as promising biomarkers for neurological disorders due to their stability in biofluids, ability to cross the blood–brain barrier, and capacity to reflect the molecular state of their cells of origin [15,60]. EVs protect their cargo from enzymatic degradation, thereby preserving disease-associated cargo that can be detected through minimally invasive sampling. Accordingly, EVs isolated from blood, cerebrospinal fluid, and saliva carry disease-relevant proteins and RNAs that mirror pathological processes in the brain and have been extensively investigated as “liquid biopsies” for neurodegenerative disorders [15,31–33].

In this context, AS represents a particularly attractive candidate for EV-based biomarker development because it is caused by the loss of a single well-defined protein such UBE3A. The recent discovery that UBE3A is physiologically released in neuron-derived EVs but absent in AS-EVs [11] provides a biological rationale for investigating UBE3A-associated EVs as potential biomarkers. If validated in patient-derived EVs, this approach could provide a rapid, minimally invasive, and mechanism-based tool for diagnosis and therapeutic monitoring.

Despite this promise, several challenges remain. First, EV isolation methods lack standardization and can significantly affect yield and purity, while the heterogeneity of EV populations complicates identification of disease-relevant EVs from CNS cell types [5]. Overcoming these technical limitations will be essential for translating EV-based biomarkers into clinical practice.

## Therapeutic Strategies for AS and Potential EV Applications

Several therapeutic strategies have been developed to restore UBE3A function in AS, including direct UBE3A delivery, engineered secretable/cell-penetrating UBE3A constructs, and lentivector-transduced hematopoietic stem cell approaches, all of which improve synaptic and cognitive phenotypes in AS mice [61–63]. More recently, reactivation of the paternal UBE3A allele has emerged as a promising therapeutic strategy, although efficacy depends on the developmental timing of intervention [64,65]. Despite these advances, current approaches remain limited by invasive delivery methods, challenges in achieving widespread and sustained UBE3A expression, the complexity of stem cell-based interventions, and critical developmental windows that may restrict therapeutic efficacy.

EVs are increasingly investigated as therapeutic tools for CNS disorders due to their biocompatibility, low immunogenicity, and ability to cross biological barriers, including the blood-brain barrier [60,66]. In addition to serving as natural carriers of proteins, lipids, and nucleic acids, EVs can be engineered to deliver therapeutic proteins, regulatory RNAs, or genome-editing systems to specific cell populations. Stem cell-derived EVs, particularly from mesenchymal stromal cells, have demonstrated neuroprotective, immunomodulatory, and synaptogenic effects in multiple preclinical neurological disease models [66,67].

Importantly, recent work by Penna and colleagues showed that administration of wild-type neuron-derived EVs rescues dendritic spine abnormalities and significantly improves learning and memory in AS mice. Although UBE3A-containing EVs may contribute to these therapeutic effects, EV-mediated rescue is unlikely to result solely from its restoration. Rather, EVs deliver a complex repertoire of proteins, mRNA, and other regulatory molecules that coordinately regulate neuronal maturation, circuit connectivity, spine formation, and synaptic plasticity [26,68]. These findings suggest that EV therapy may simultaneously correct multiple pathological pathways disrupted by UBE3A deficiency.

Additionally, EVs offer versatility as engineerable delivery vehicles. They can be modified to carry specific RNAs, proteins, or genome-editing tools such as CRISPR-Cas systems [69,70], while surface engineering can enhance uptake by defined neuronal or glial populations. In principle, disorders linked to synaptic dysfunction or proteostatic imbalance, such as those involving UBE3A deficiency, could benefit from EV-mediated restoration of regulatory proteins or RNA networks directly within the CNS.

Despite their considerable promise, several challenges must be overcome before EV-based therapies can be translated into clinical practice. These include large-scale EV production, purification and characterization of heterogeneous EV preparations, optimization of biodistribution and targeting, and establishment of standardized potency and dosing criteria [5,71]. In parallel, comprehensive long-term safety studies will be essential to evaluate EV biodistribution, persistence and potential adverse effects following repeated administration. Furthermore, defining the molecular determinants underlying EV therapeutic activity will be critical for identifying reliable potency markers and generating optimized EV formulations suitable for clinical translation. Addressing these challenges will be essential for realizing the full therapeutic potential of EVs.

Importantly, evidence supporting EV-based therapies in AS is currently limited to animal models. While this model improved our understanding of the molecular mechanisms underlying the disease, the translation to humans remains uncertain. Human induced pluripotent stem cell (hiPSC)-derived neurons and brain organoids generated from AS patients recapitulate key molecular and functional features of the disorder [72–74] and provide a valuable platform to evaluate EV cargo, uptake, and therapeutic efficacy in a human context. Recent studies have demonstrated that EVs can rescue disease-associated phenotypes in hiPSC-derived neurons from patients with neurodevelopmental disorders [75,76], highlighting the utility of these models for validating therapeutic efficacy and mechanisms. In the future, AS patient-derived hiPSC models may facilitate the understanding of EV pathogenesis, biomarker discovery, and provide critical information for the development of engineered EV-based therapies.

## Concluding Remarks

EVs represent a fundamental mechanism of intercellular communication in the CNS, integrating neuronal activity, glial signaling, and immune responses to regulate synaptic plasticity, metabolic homeostasis, and neural circuit functions. Thus, disruption of EV-mediated communication is increasingly recognized as a common mechanism contributing to both neurodegenerative and neurodevelopmental disorders.

The emerging evidence linking EV dysfunction to synaptic and cognitive deficits in AS represents an important conceptual advance, suggesting that AS pathophysiology extends beyond intracellular consequences of UBE3A deficiency to include impaired intercellular communication. Current findings support a model in which UBE3A loss disrupts endosomal-lysosomal trafficking, leading to abnormalities in EV biogenesis, cargo composition, secretion, and uptake, ultimately compromising neuronal connectivity and circuit maturation.

Future studies should define the molecular mechanisms linking UBE3A deficiency to EV dysfunction and determine the extracellular functions of UBE3A. In particular, investigating how UBE3A, LAMTOR1, and TRPML1 coordinate endolysosomal trafficking and EV dynamics may provide important insights into the mechanisms underlying Angelman syndrome and other neurodevelopmental disorders.

The therapeutic potential of EVs requires further investigation. Although the findings from Penna et al. suggest that EV delivery of UBE3A rescues dendritic spine maturation and cognitive deficits in AS mice, the molecular determinants underlying these effects remain to be identified. The observed rescue may also involve additional EV cargoes, including proteins and regulatory RNAs that contribute to neuronal maturation, connectivity, spine formation, and synaptic plasticity [26,68]. Notably, several UBE3A substrates, including Arc and PEG10 have been identified in EVs and are known to regulate intercellular communication and neuronal plasticity [46,77], raising the possibility that restoration of EV-mediated signaling, rather than replacement of a single protein, provides additional therapeutic benefit [78,79]. Future studies using human cellular models and patient-derived systems will be essential to validate these mechanisms and facilitate translation of EV-based therapies into clinical applications.

Collectively, the studies reviewed here support a paradigm shift in our understanding of Angelman syndrome. Rather than viewing AS solely as a cell-autonomous disorder caused by intracellular UBE3A deficiency, accumulating evidence indicates that impaired EV-mediated communication is an integral component of disease pathophysiology. Although emerging evidence supports this non-cell-autonomous framework, direct studies are required to establish the causal role of EV-mediated intercellular communication in AS and to define the molecular pathways involved. This emerging perspective not only provides new mechanistic insight into neural circuit dysfunction but also identifies EVs as a promising avenue for the development of next-generation therapies for Angelman syndrome.
